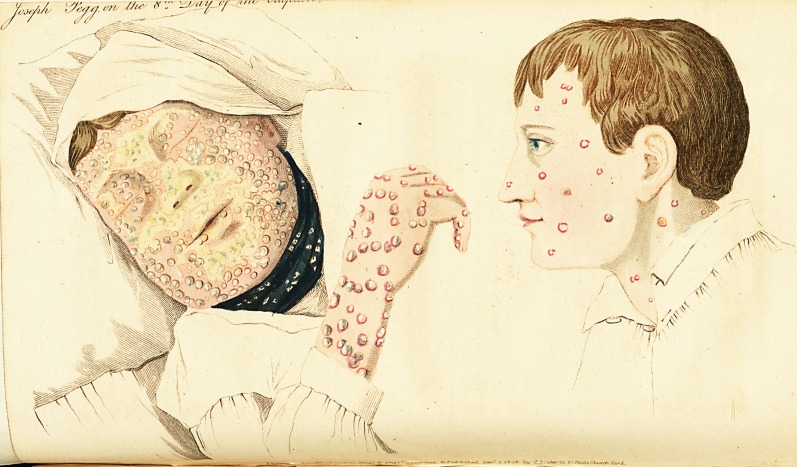# Observations on an Epidemic Varioloid Disease, Lately Witnessed in the County of Derby

**Published:** 1818-12

**Authors:** Thomas Bent


THE LONDON

### Medical and Physical Journal.

6 OF VOL. XL.] DECEMBER, 1818. [NO. 238.

"For many fortunate discoveries in medicine, and for the detection of nume-"rous errors, the world is indebted to the rapid circulation of Monthly "Journals; and there never existed any work to which the Faculty in "EUROPE and AMERICA were under deeper obligations than to the "Medical and Physical Journal of London, now forming a long, but an "invaluable, series."—RUSH.

*For the London Medical and Physical Journal.*

### *Observations on an Epidemic Varioloid Disease, lately witnessed in the County of Derby.* By THOMAS BENT, M.D.

IN the Reports of the National Vaccine Establishment for 1817, it is admitted, that "accounts are occasionally received of failures in vaccination;" and, in the Report of Diseases which is usually made in one of the monthly Medical Journals, it is stated, that "accounts from the country of the occurrence of small-pox, in a mild form, after vaccination, continue to be received." I have waited anxiously with the hope and expectation that some information of a more precise description would be given to the public on this very interesting subject. I am sorry to say, it has happened to the medical practitioners of this town and neighbourhood to witness the occurrence of a severe eruptive disease after vaccination, and likewise, independent of it, in many hundred instances; and the disease is at this moment prevailing very extensively.

In the spring of 1816, several cases of small-pox were reported to have occurred after vaccination, at Quarndon, a village about two miles from Derby; and one or two individuals were said to have died of it. These rumours excited much public attention, and induced many of the neighbouring medical men to visit the place, and examine the nature of the disease. They differed in their opinions; the majority regarding the symptoms to be those of an aggravated form of chicken-pox, and some considering it to be a mild form of small-pox after vaccination. Other similar cases were met with in a few instances, but little more was heard of the disease after the autumn. In the following spring, it showed itself again at Breadsall, Smalling, Spondon, Heanor, and some other districts within a few miles of the town; and in some of these places the majority of the children and young people were afflicted with it. It declined again in the autumn; but re-appeared more generally than before during the late spring, and is yet very prevalent, in the town, to an extent not before manifested.

Several cases have been brought to the Infirmary, which have afforded opportunities for more minute examination into the particular character of the eruption, than can usually be obtained in other situations. The appearances which have been presented in different individuals have been extensively varied, and I can venture to affirm, that every gradation imaginable, between the mildest form of chicken-pox and a severe small-pox, has been observed. If I were to make any general conclusion on this part of the subject, it would be, that, in the majority of examples, the disease has exhibited those particular appearances which Dr. Wilson has described as occurring in the instances where the influence of cow-pox has modified small-pox:—"The pocks have a vesicular character, and often a small horny appearance, and pass through the usual stages; but, instead of proceeding to full suppuration, they begin to subside, and dry away on the sixth day from the commencement of the eruption." A very curious and important feature in the history of this disease is, that the general symptoms and the particular character of the eruption *is the same in those persons who have never had the cow-pox, as in those who have passed through that disease satisfactorily.* I have this day seen two children of the same family, one a year older than the other,—the former had been vaccinated, and the scar on the arm was of the usual appearance; the younger one had never been inoculated. Both became unwell about the same time, and the eruption, after slight febrile symptoms, showed itself on each on the same day. The pustules were distinct in both, but more numerous on the elder, who was the most unwell of the two. I first saw them on the fifth day of the eruption, when some of the pocks were filled with a semi-transparent fluid, and others had become dry and formed a crust. At the same period, in the child which had not been vaccinated, the eruption had a papular appearance in some places, without any show of fluid; and the mother said, "it had been more out, but had gone in again." In other parts, particularly the arms, it presented a similar character to that of the other child. Both were quite well on the ninth day of the eruption, and the scabs had mostly disappeared. The following case, which was one of the most severe and most prolonged of any I have witnessed, was treated in the Infirmary. The subject of it was leading a very irregular life at the time of the attack, had been exposed to bad weather, had lain out of doors two or three nights, and was labouring under pulmonic inflammation when seized with the first symptoms of eruptive disease.

April 9th, 1818.—Mary Miller, aged 20, was admitted yesterday evening; says, that on Sunday night (April 5th,) she perceived some eruption on her arms, which increased and appeared all over her during the next day; that she had felt unwell three or four days previously; that she had been in a house with two children who were supposed to have small-pox, and that one died. The pocks, which are numerous and coherent on the face, but distinct on the body and limbs, are now in different states: the greater part are flat, have but little inflammation at the base, and contain a transparent fluid; and there are many which are appearing as red points upon the skin. She has been subject to a cough for some time previous to the present attack, which was severe yesterday, but is better to-day. The nurse says, she appeared hot, restless, and delirious, in the night: she is now cool, has a moist tongue, and her pulse is round, soft, and about ninety.

10.—The pustules, which contained lymph yesterday, are to-day flat and indented in the middle; on many there is a crust. The inflammation, which appeared yesterday about the base of some of the pustules, is now gone; and those which were small, and like red pimples, have a head upon them, and contain a limpid fluid. There is some swelling of the face, and the eyes are partly closed.

13.—Most of the pustules on the face are dry and hard on the top; some contain fluid, which is become thicker and more opaque; those on the limbs still contain a perfectly limpid fluid, and are free from inflammation. There is eruption in the internal fauces, which occasions impeded respiration, hoarseness, and ptyalism.

14.—Respiration easier; the greater part of the pustules on the limbs are becoming dry; in many, there is an opaque fluid.

16.—The scabs are separating in the face, and the pustules are become dry generally, in other parts; in a few, there was transparent lymph so late as yesterday.

17.—Improves in strength, and has a good appetite. The scabs are mostly separated in every part, and there is no indentation left in the skin, except in a few instances on the face, where a slight superficial pitting appears, particularly about the chin.

20.—Complains of soreness in her throat and difficulty of swallowing. There are scabs in a few places not yet separated.

The woman remained in the house two or three weeks longer, but nothing in her complaints afterwards affected the subject of her eruptive disease.

On the 17th, I visited a child at Alroston, two miles from Derby, at a house where Mary Miller had been put to-bed the day but one before she was brought to the hospital. This child slept in the bed from which she had been removed on the same night, without the linen being changed. In a few days afterwards, she became slightly unwell, and an eruption appeared in another day or two. The pustules, which were very few in number, and principally on the back, were small, and contained a little transparent fluid; some, at the same time, being covered with a dry brown crust. The disease terminated about the sixth day of the eruption, and the child was well before the woman from whom she received the infection.

Three or four medical friends, who visited this child, did not hesitate in pronouncing the case to be chicken-pox; and it had certainly all the characters of a mild attack of that complaint. It may be well to observe, that no one in the village was affected with chicken-pox at the time, and the child had not been in any situation where there was the least probability of its receiving infection, except from Mary Miller.

A young man, an apprentice to one of the surgeons to the hospital, after being several times about the bed of Mary Miller, became unwell, and broke out. The eruption remained three or four days only, and the disease was, in all other respects, similar to that of the child at Alroston. Neither the woman nor the child had had the vaccine disease; the young man had been vaccinated, and passed through the disease regularly.

Joseph Pegg, a boy about 14 years old, who was in the hospital, and had just recovered from dropsy, after having had febrile symptoms for two or three days, on the 1st of May, in the evening, perceived two or three small pimples on his chin; and on the following morning there were many on other parts of his face. On the 3d, they were more numerous, and many appeared on the body and limbs. One or two on the chin contained fluid. He had a quick pulse, and considerable fever; said, he had been inoculated for cow-pox without effect. On the 5th, the pocks contained fluid, were flat, and had a slight indentation, about the size of a pin-head, in the centre. There was much swelling and general redness over the face, and the eyes were closed.

Some parts of the eruption had the appearance of being more recent, and looked like red pimples. On the 7th, the swelling of the face was very considerable; and there was a slight circumscribed redness about the pocks on the limbs. Those which first appeared on the face were become dry, the scab having more of a yellow than a brown appearance; others, on the same part, still contained a fluid perfectly limpid. On the 10th, the swelling of the face had subsided, and the eruption generally was dry and scabby. On the limbs, the pocks were large, and still contained, in some a transparent fluid, in others it was opaque; and the eruption on those parts had a pearly appearance; so much so, that one individual, who saw it, remarked, that the boy looked as if he were covered over with small pearl buttons. The separation of the scabs left a peculiar appearance on the skin: instead of indentations, there were numerous elevations, without discoloration, and the skin looked as if covered with a colourless tubercular form of eruption.

The daughter of an eminent surgeon of this place, who had the vaccine disease satisfactorily fourteen years ago, is at the present moment labouring under the complaint. The progress of her symptoms, and the appearances of the eruption, bear a very close resemblance to those of Joseph Pegg; and her mother, to whom a drawing taken from him was shown, at first believed it was intended for her daughter, and exclaimed "How like it is!"

There is not a single pit or depression left in the face of Joseph Pegg, nor in any other part; which tends to prove that the pocks never underwent the suppurative process, although the swelling and inflammation about the face were so great. An additional proof of pus being rarely formed, is derived from the circumstance of the peculiar odour, which is usually emitted in genuine small-pox, being scarcely ever perceived.

On the 5th of May, Matilda Dixon, a child 17 months old, was inoculated with lymph taken from Mary Miller, a fortnight after the commencement of the eruption. She sickened in a few days, and became fretful and slightly feverish; an eruption, thinly dispersed, appeared. The pocks were small, soon formed a head, and dried about the fourth or fifth day. She had not been inoculated with vaccine virus previously. Several other children have been inoculated. In some, the result has been similar to that in the instance of Matilda Dixon; in others, there have been more fever, more eruption, and much more severe and prolonged illness; but the pocks have generally preserved one character: they have been vesicular, and have become dry about the sixth day, without going on to suppuration. The appearances presented in a great variety of cases have, however, been sufficiently varied to give rise to the supposition that the contagions of variola and varicella might both be prevailing; but the experiments which have been made in the way of inoculation prove, pretty satisfactorily, that one contagion gives rise to the whole, and that the differences are owing, in all probability, partly to early treatment, but mainly to constitutional peculiarity. Had both diseases been prevalent, one should expect to meet with the occurrence of one after another in the same individual, in some instances; but this has never happened in any case within my knowledge.

It is rather a curious circumstance that, in the village of Spondon, where the greater part of the children and young people have had the disease, the family of a most respectable surgeon, who resides there, (twelve or thirteen in number,) who have been exposed to its contagion in every possible way, have not one of them been afflicted; and, consequently, he has been charged by the vulgar with having inoculated their families with bad matter, at the same time that he procured good for his own. With varicella, the disease of which we are treating has many symptoms in common, and numerous examples appear identical with it; but, in very many others, it is more severe, of longer duration, and more fatal in its consequences, than I have ever witnessed or seen described in that complaint.

The great fickleness and inconstancy which the disease presents, have at times led me to consider, whether modern physicians have not erred in making variola and varicella distinct diseases; and whether Morton and Sydenham, and their contemporaries, who considered them as varieties merely of the same disorder, may not prove to be the more accurate observers. The disease has proved fatal in many instances amongst the lower orders, who unfortunately retain the old prejudice respecting the necessity of encouraging eruption by means of flannel and fermented liquors. Under better treatment, it has also occasioned some deaths. One in-patient at the Infirmary, who had previously had the vaccine disease, and two out-patients, one of whom had also had it, have been victims to it.

I have hitherto been a steady advocate for vaccination, a firm believer in its protecting influence; and, after the trials which have often been successfully made by myself and others to prove its power in arresting the attacks of small-pox, I am much astonished at the results of recent observations. I am informed, the same disease has been frequent in

Nottinghamshire and Staffordshire; but conceive, from some cause or other, it has prevailed more extensively here than elsewhere. Can this be owing to an imperfect vaccine virus having been used in this neighbourhood? Much, I believe, has been supplied from the Infirmary, where inoculation has been regularly performed, and for which the Vaccine Establishment have repeatedly supplied the virus.

May not the contagion of small-pox, after having been introduced into the system of an individual, or a succession of individuals, who are under the protection of vaccine disease, have its qualities so changed as to allow of its propagating a disease in others, free from vaccine influence, similar to that by which it was itself produced? If this conjecture should prove well-founded, the prevailing disease may be deemed a new variety of small-pox.

The conclusions which I am inclined to make from the facts which have hitherto been presented to me, are—

1st. That the disease which I have described is not the genuine small-pox.

2nd. That it differs from chicken-pox in duration, severity, and consequences.

3d. That it is not merely small-pox modified by vaccine influence, because the symptoms are the same in those who have not had vaccine disease as in those who have.

4th. That vaccination has an imperfect, if any, influence in arresting its attack.

In several instances, the disease has attacked children who have recently recovered from cow-pox.

The two drawings were made by my friend Mr. Bennett, a very assiduous and attentive surgeon of this place, who has given much attention to the peculiarities of the complaint. They are very exact representations of the appearance of the skin in the case of Joseph Pegg, on the eighth day of the eruption; and of another person who had the disease in a milder form, on the fifth day. In both, the pocks are seen in various states; some having formed a hard crust, and others having newly formed a head.

*Derby; October 14th*, 1818.

---

*For the London Medical and Physical Journal.*

## Figures and Tables

**Figure f1:**